# The changes in psychological symptoms of COVID-19 patients after “re-positive”

**DOI:** 10.3389/fpsyt.2022.1010004

**Published:** 2022-10-10

**Authors:** Xing Wang, Qinyi Fan, Yunyue Li, Junjian Xiao, Yanyan Huang, Tiantian Guo, Hongguang Chen, Mengqian Li

**Affiliations:** ^1^School of Life Sciences, Nanchang University, Nanchang, China; ^2^Clinical Medical Experiment Center, Nanchang University, Nanchang, China; ^3^The First Psychiatric Hospital of Harbin, Harbin, China; ^4^Queen Mary College, Nanchang University, Nanchang, China; ^5^Department of Psychosomatic Medicine, The First Affiliated Hospital of Nanchang University, Nanchang, China; ^6^NHC Key Laboratory of Mental Health (Peking University), Peking University Sixth Hospital, National Clinical Research Center for Mental Disorders, Peking University Institute of Mental Health, Beijing, China; ^7^Department of Psychosomatic Medicine, Gao Xin Hospital of The First Affiliated Hospital of Nanchang University, Nanchang, China

**Keywords:** COVID-19 patients, re-positive, anxiety, depression, insomnia

## Abstract

**Background:**

Previous studies have showed that individuals infected with COVID-19 were more likely to report psychological symptoms. However, little is known about the changes from testing positive to negative to positive again.

**Methods:**

This survey was conducted through the questionnaires including the 7-item Generalized Anxiety Disorder (GAD-7), the 9-item Patient Health Questionnaire (PHQ-9), as well as the Self-Rating Scale of Sleep (SRSS) to explore the psychological status of COVID-19 and re-positive cases.

″re-positive″ is defined as a positive RT-PCR test at any time during the recovery period after testing negative.

**Results:**

A total of 94 COVID-19 patients presented the prevalence rates of anxiety, depression, insomnia, and any of the three psychological symptoms being 26.6, 8.6, 12.8, and 31.9%, respectively. Among these, 32 cases were re-tested positive during the recovery period, with the prevalence rates of anxiety, depression, insomnia, and any of the three psychological symptoms being 21.9, 18.7, 31.2, and 37.5%, respectively. The psychological status after re-positive showed a significant decrease in anxiety (*P* = 0.023), an increase in depression, and a significant rise in insomnia (*P* = 0.035). For those with no psychological symptoms during initial-positive, after re-positive, 5.88% reported anxiety, 5.88% reported depression, and 11.76% reported insomnia. For those who experienced only anxiety symptoms during initial-positive, after re-positive, 33.3% reported depression, and 33.3% reported insomnia.

**Conclusions:**

Our findings encompassed the urgent concern for anxiety in initial-positive COVID-19 patients, depression in re-positive COVID-19 patients, and insomnia in both initial and re-positive patients, hence enabling targeted interventions for appeasing the psychological burden of COVID-19 patients.

## Introduction

There is growing evidence of studies associating COVID-19 survivors with increased psychological symptoms. It's reported that symptoms aroused by COVID-19 infection, side effects of treatment, concerns about sequelae, social isolation, and stigma all partook to a higher risk of psychological symptoms in COVID-19 patients (1–3). Previous studies recounted that the prevalence of cases with post-infection anxiety ranges from 6.5 to 63% (4–6). In studies implicating both hospitalized and non-hospitalized patients, the prevalence rates for depression and insomnia range between 12 and 48 and 2–63%, respectively (4, 7, 8). Psychological symptoms and implications related to a COVID-19 infection comprise both acute and long-term consequences. In addition, the re-positive rate was found to be correlated to illness severity, according to the Acute Physiology and Chronic Health Evaluation II (APACHE II) severity-of-disease classification system, and the confusion, urea, respiratory rate, and blood pressure (CURB-65) score. Since the physical condition may worsen after re-positive, what will take place in the psychological status of COVID-19 patients remain unknown. This study aims to explore changes in the severity of psychological symptoms and the mutual changes in anxiety, depression, and insomnia in re-positive COVID-19 patients.

## Methods

### Participants and procedure

A total of 94 initially confirmed COVID-19 patients were enrolled from Bayan County and Pingfang District of Harbin City, in Heilongjiang Province from September 23, 2021, to December 31, 2021. Located in the northern part of Northeast China, Harbin is the capital city of Heilongjiang Province with a resident population of approximately 9.8 million. Since the outbreak of the COVID-19 pandemic in 2020, Harbin has been a low-prevalence city with two small-scale indigenous outbreaks.

These patients were diagnosed and treated fulfilling the criteria of the 8th version of the Guidelines on the Diagnosis and Treatment of COVID-19 by the National Health Commission of China. Confirmed patients with COVID-19 were treated at one of the designated hospitals, the Harbin Infectious Diseases Hospital, which was a dedicated hospital for COVID-19 patients. Once the RT-PCR test turned negative after the treatment, patients were transferred to designated Harbin Second Hospital for rehabilitation. Paper-and-pencil questionnaires were utilized to investigate the psychological symptoms of all COVID-19 patients transferred to Harbin Second Hospital. A total of 94 respondents were enrolled except for two children. ″Re-positive″ is interpreted as a positive RT-PCR test at any time during the 14 days or 14+7 days recovery period in the Harbin Second Hospital. Of the 94 COVID-19 negative cases, 32 were re-positive during the recovery period. The re-positive patients will be re-transferred to Harbin Infectious Diseases Hospital for treatment until their tests turn negative and then transferred back to Harbin Second Hospital for recovery. Both the initial-positive patients and re-positive patients were surveyed within 2 days of admission to the Harbin Second Hospital. All the 32 re-positive cases received the two questionnaires at intervals of more than 2 weeks. This study was approved by the Research Ethics Committee of the First Affiliated Hospital of Nanchang University (Ethical number: 2020-051).

### Measures

Anxiety, depressive symptoms, and insomnia were assessed using the 7-item Generalized Anxiety Disorder (GAD-7) (9), the 9-item Patient Health Questionnaire (PHQ-9) (10), and the Self-Rating Scale of Sleep (SRSS) (11) in Chinese version, separately. Each item on the GAD-7 and PHQ-9 was gauged with a four-point Likert scale (0= “not at all” to 3 = “extremely”) to infer the severity of a particular symptom within the past 2 weeks. There are 10 items in total, and each item has five levels (1–5). The higher the score, the more severe the sleep problem. The cut-offs to screen for possible positive cases of anxiety, depression and sleep disorders were a GAD-7 score ≥5, a PHQ-9 score ≥5, and an SRSS score ≥23, respectively. The Cronbach's α for self-reported GAD-7, PHQ-9, and SRSS for COVID-19 patients in the first investigation were 0.92, 0.78, and 0.91, respectively. For COVID-19 re-positive patients in the second survey, the Cronbach's α for GAD-7, PHQ-9, and SRSS were 0.96, 0.84, and 0.92, respectively.

### Statistical analyses

Descriptive analyses were executed based on frequencies for all variables, including demographic data, as well as factors linked with the risk of COVID-19 cases, which were applied by the exact probability tests as the low frequencies. The paired samples *t*-test or Chi-squared test was conducted to spot the differences in psychological status of the 32 patients pre-and post-re-positive. Data were analyzed employing the statistical software IBM SPSS version 22 (SPSS Inc., Chicago, IL, USA), and figures were drawn by GraphPad prism 9 (GraphPad Software, Inc., San Diego, CA). All statistical analysis was performed with a *P*-value < 0.05 using a two-tailed test deemed statistically significant.

## Results

### Sociodemographic characteristics

A total of 94 initially confirmed COVID-19 patients were implicated, with 55 females and 39 males, of which 32 were re-tested positive for COVID-19 during rehabilitation. The sociodemographic characteristics of 94 patients were as follows: 58.5% female, 50.0% older than the age of 45 years, 16.0% with at least one underlying disease, 84.0% quarantined for more than 15 days, 76.6% married, 83.0% with high school or less, 24.4% with no source of revenue.

### The prevalence rates of anxiety, depression, and insomnia symptoms among COVID-19 patients

The prevalence of anxiety, depression, insomnia, and any of the three psychological symptoms in initially confirmed 94 COVID-19 patients were 26.6, 8.6, 12.8, and 31.9% (see Table 1), respectively. For the 32 re-positive cases, the prevalence rates were 43.8, 12.5, 21.9, and 46.9% (data not shown) in the initial-positive, respectively. The prevalence rates after re-positive were 21.9, 18.7, 31.2, and 37.5 %, with increases of −50.0% (χ^2^ = 6.411, *P* = 0.001), 50.0% (χ^2^ = 0.117, *P* = 0.732), 42.9% (χ^2^ = 6.733, *P* = 0.009) and −20.0% (χ^2^ = 16.737, *P* < 0.001). Most of the initial COVID-19 cases screening positive for anxiety, depression, and insomnia showed mild symptoms. Nevertheless, there was an increase in the proportion of cases with severe anxiety and moderate insomnia in COVID-19 patients after re-positive. Notably, an impressive proportion of COVID-19 patients with anxiety symptoms was observed in cases with medical history than those without (*P* < 0.05) (see Table 2). Moreover, a higher proportion of re-positive patients with insomnia was viewed over the age of 45 years (*P* < 0.05) (see Table 3). We also compared the demographic characteristics of 32 re-positive cases and the remaining 62 cases without re-positive cases and found that there was no statistical difference in demographic characteristics between the two groups except that the proportion of cases with a medical history was higher in re-positive cases than that in the other group (*P*=0.006) (see Supplementary Table 1).

**Table 1:** The prevalence and severity of anxiety, depression, and insomnia among COVID-19 patients.

	**Anxiety**		**Depression**		**Insomnia**		**Anyone^†^**	
	**COVID-19 cases** **(*N* = 94)** ***n* (%)**	**Re-positive cases** **(*N* = 32)** ***n* (%)**	**COVID-19 cases** **(*N* = 94)** ***n* (%)**	**Re-positive cases** **(*N* = 32)** ***n* (%)**	**COVID-19 cases** **(*N* = 94)** ***n* (%)**	**Re-positive cases** **(*N* = 32)** ***n* (%)**	**COVID-19 cases** **(*N* = 94)** ***n* (%)**	**Re-positive cases** **(*N* = 32)** ***n* (%)**
Normal	69 (73.4%)	25 (78.1%)	86 (91.4%)	26 (81.3%)	82 (87.2%)	22 (68.8%)	64 (68.1%)	20 (62.5%)
Mild	20 (21.3%)	3 (9.4%)	6 (6.4%)	4 (12.5%)	12 (12.8%)	8 (25.0%)	29 (30.9%)	10 (32.3%)
Moderate	3 (3.2%)	1 (3.1%)	1 (1.1%)	1 (3.1%)	0	2 (6.2%)	4 (4.3%)	3 (9.4%)
Severe	2 (2.1%)	3 (9.4%)	1 (1.1%)	1 (3.1%)	0	0	3 (3.2%)	3 (9.4%)

† The total rate is more than 100.0% since comorbidity is indicated here.

**Table 2:** The demographic distribution of psychological symptoms among initially confirmed COVID-19 patients (*N* = 94).

**Characteristics**	**Group**	**Anxiety**		**Depression**		**Insomnia**	
		***N* (%)**	***P***	***N* (%)**	***P***	***N* (%)**	***P***
Gender	Male	8 (20.5%)	0.345	1 (2.6%)	0.134	3 (7.7%)	0.348
	Female	17 (30.9%)		7 (12.7%)		9 (16.4%)	
Age	< 45	7 (14.9%)	0.065	3 (6.4%)	0.714	4 (8.5%)	0.355
	≥45	18 (38.3%)		5 (10.6%)		8 (17.0%)	
Medical history	No	17 (21.5%)	0.022	5 (6.3%)	0.113	8 (10.1%)	0.096
	Yes	8 (53.3%)		3 (20.0%)		4 (26.7%)	
The total duration of isolation, day	7–15	6 (40.0%)	0.125	2 (13.3%)	0.609	1 (6.7%)	0.683
	>15	19 (24.1%)		6 (7.6%)		11 (13.9%)	
Marital status	Married	20 (27.8%)	0.786	4 (5.6%)	0.084	8 (11.1%)	0.466
	Others	5 (22.7%)		4 (18.1%)		4 (18.1%)	
Educational level	High school or less	22 (28.2%)	0.546	7 (9.0%)	1.000	10 (12.8%)	1.000
	Undergraduate degree/college	3 (18.8%)		1 (6.3%)		2 (12.5%)	
Source of income	No	3 (13.0%)	0.217	0 (0)	0.276	3 (13.0%)	0.901
	Not sure	3 (25.0%)		1 (8.3%)		2 (16.7%)	
	Yes	19 (32.2%)		7 (11.9%)		7 (11.9%)	

**Table 3:** The demographic distribution of psychological symptoms among re-positive confirmed COVID-19 patients (*N* = 32).

**Characteristics**	**Group**	**Anxiety**		**Depression**		**Insomnia**	
		***N* (%)**	***P***	***N* (%)**	***P***	***N* (%)**	***P***
Gender	Male	3 (30.0%)	0.648	3 (30.0%)	0.346	4 (40.0%)	0.683
	Female	4 (18.2%)		3 (13.6%)		6 (27.3%)	
Age	< 45	1 (8.3%)	0.212	1 (8.3%)	0.370	0(0)	0.004
	≥45	6 (30.0%)		5 (25.0%)		10 (50.0%)	
Medical history	No	5 (22.7%)	1.000	4 (18.2%)	1.000	6 (27.3%)	0.683
	Yes	2 (20.0%)		2 (20.0%)		4 (40.0%)	
The total duration of isolation, day	7–15	N/A	N/A	N/A	N/A	N/A	N/A
	>15	7 (21.9%)		7/32(21.9%)		10 (31.25%)	
Marital status	Married	6 (25.0%)	0.646	5 (20.8%)	1.000	8 (33.3%)	1.000
	Others	1 (12.5%)		1 (12.5%)		2 (25.0%)	
Educational level	High school or less	7 (25.9%)	0.560	6 (22.2%)	0.555	9 (33.3%)	1.000
	Undergraduate degree/college	0(0)		0 (0)		1 (20.0%)	
Source of income	No	1 (14.3%)	1.000	1 (14.3%)	1.000	2 (28.6%)	1.000
	Not sure	0 (0)		0 (0)		1 (50.0%)	
	Yes	6 (26.1%)		5 (21.7%)		7 (30.4%)	

### The severity changes of the three psychological symptoms after re-positive

**Figure 1:**
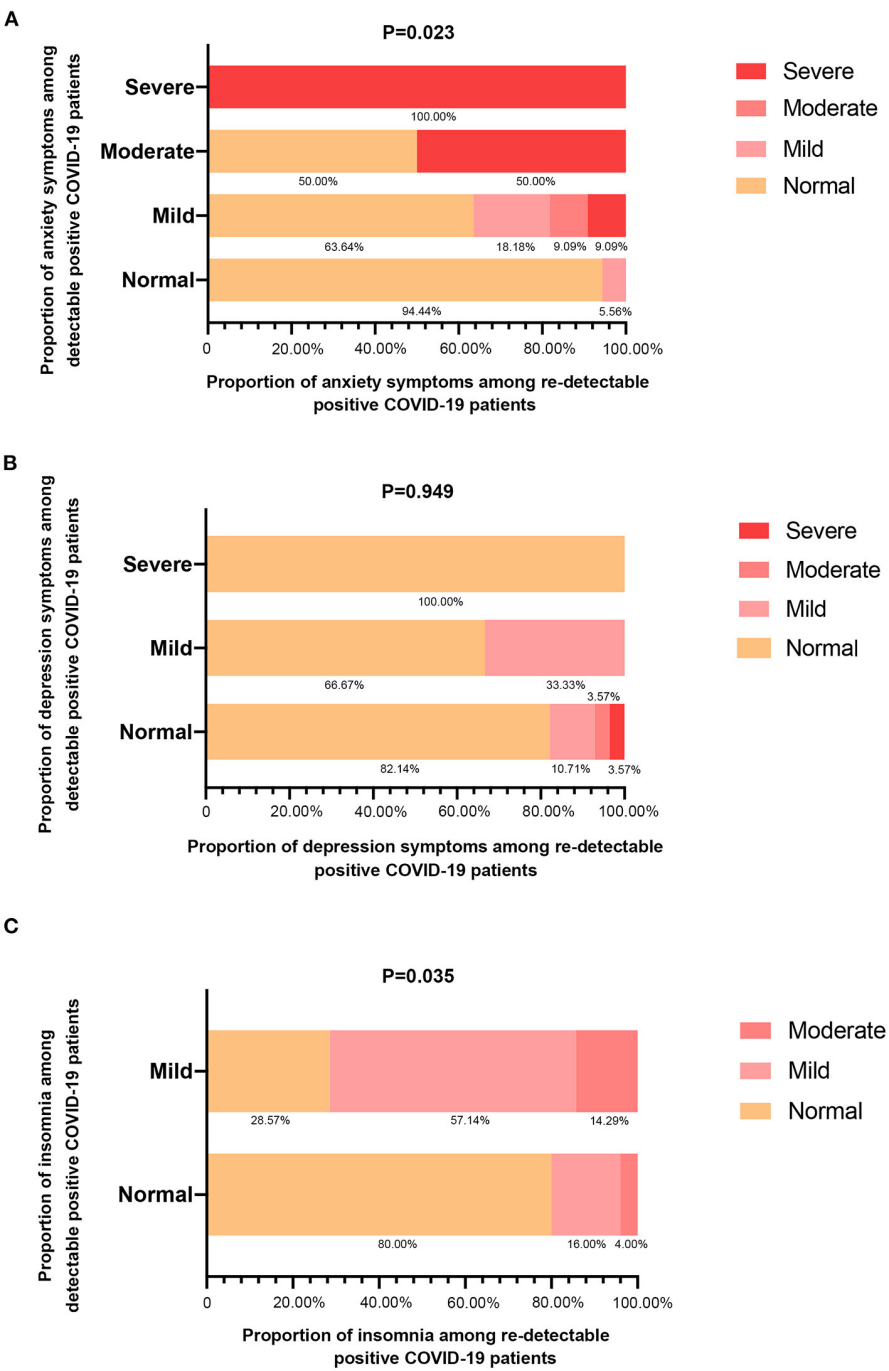
Stacked bar charts illustrated the conversion of psychological status after re-detectable positive for COVID-19 patients following anxiety symptoms **(A)**, depression symptoms **(B)**, and insomnia **(C)**. Statistical comparison was obtained by the paired samples *t*-test for the alteration levels of all mental health problems.

Furthermore, the paired samples *t*-test comparing the consistency assessment of COVID-19's severity between 32 initial-positive and re-positive, reflected a general decrease in anxiety (*P* = 0.023), with most of the re-positive patients who initially exhibited mild or moderate anxiety symptoms becoming normal (Figure 1A); with a slight but not significant increase in depression symptoms (*P* = 0.949), 66.67% of initially mild and 100.00% of initially moderately cases altering into normal, while 17.85% of initially normal cases presented with depression (Figure 1B). Moreover, these patients withstood a significant increase in insomnia after re-positive (*P* = 0.035), as 20.00% of initially normal patients suffered from insomnia and 14.29% of initially mild insomnia transformed into the moderate level (Figure 1C).

### The mutual changes of three psychological symptoms after re-positive

In addition, we compared the conversion rates among the three psychological symptoms between initial-positive and re-positive. Results demonstrated that for those who had no psychological symptoms during initial-positive, after re-positive, 5.88% reported anxiety problems, 5.88% reported depression symptoms, and 11.76% reported insomnia. For those who were only positive for anxiety symptoms during initial-positive, after re-positive, 33.3% reported depression, and 33.3% reported insomnia (see Table 4).

**Table 4:** The mutual changes of psychological symptoms after re-positive.

**Initial-positive cases**	**Total**	**Re-positive cases**		
		**Anxiety + *N* (%)**	**Depression + *N* (%)**	**Insomnia + *N* (%)**
Anxiety-, Depression-, Insomnia-	17	1 (5.88%)	1(5.88%)	2 (11.76%)
Anxiety+, Depression -, Insomnia-	6	3 (50.00%)	2 (33.33%)	2 (33.33%)
Anxiety -, Depression +, Insomnia-	0	N/A	N/A	N/A
Anxiety -, Depression -, Insomnia+	1	0 (0)	0 (0)	1 (100.00%)

(+) self-report positive, (-) self-report negative.

## Discussion

In this study, we analyzed the psychological effects of the re-positive test on COVID-19 patients by comparing the changes in psychological symptoms between the initial-positive and re-positive. The detection rate of cases with psychological symptoms in this study was slightly lower than those announced in previous studies. A study from the late COVID-19 implied that the prevalence of depression and anxiety symptoms was 49.06 and 56.60% in COVID-19 patients (12). Additionally, a meta-analysis of 31 studies of COVID-19 patients revealed a pooled prevalence of 47% for anxiety, 45% for depression, and 34% for insomnia (8). The differences may be pertained to the epidemic control during the study period, the progress of vaccine development and vaccination, COVID-19 treatment, measurement, and the severity of the included subjects. Since these studies were performed at the early stages of the outbreak, when little was known about the virus, an effective vaccine, or a specific therapeutic agent, the uncertainty bestowed to high-level patient stress and fear of disease. Furthermore, the information overload generated by constant news media reports about deaths of infected cases contributed to patients' vulnerability to depression, and even negative thoughts after infection (13).

The stressors for COVID-19 patients were the disease itself, treatment regimen, and worries about family health, resulting in changes in mood, sleep, behavior, etc. The gradual adaptation process was reflected in COVID-19 patients' attitudes upon admission, from uncertainty about the disease to anticipation and suspicion of the examinations, to confrontation and acceptance following the diagnosis, which ultimately culminated in gratitude for the experience (14). This study also identified a significant reduction in symptom burden associated with depression and anxiety over time with hospitalization (15). However, cases who had been gradually improving physically and psychologically with the treatment appeared to experience changes in their psychological state after experiencing a re-positive result. Our findings indicated a general decrease in anxiety symptoms, and a slight increase in depressive symptoms of COVID-19 patients after re-positive. Notably, the sleep status of the patients deteriorated considerably, with a significant increase in insomnia. This might be explained by the disrupted discharge expectations and psychological distress caused by all kinds of uncertainties after re-positive.

For re-positive COVID-19 cases, the considerable worsening of insomnia earned attention. Being in prolonged hospitalization, their sleep habits might be affected by reduced physical activity, psychological stress, lack of a regular work schedule and social activities, changes in living conditions, etc. (16). Furthermore, due to the absence of other recreational pastimes and a rigorous schedule, COVID-19 patients might also devour more time on electronic devices and have particular potential to experience altered biorhythms with late bedtimes and late wakeups during hospital admission (17). Previous research has also reported that spending more time on electronic devices before falling asleep affects sleep quality (18). In addition, re-positive patients are likely to be more apprehensive about their health status and experience greater fear of sequelae. With extended treatment periods, there may also be a feeling of more loneliness. These presumably explain the deterioration of sleep status in re-positive patients. The social and family burden of cases over 45 years old were relatively heavy, and the problem of insomnia in this group after re-positive was more prominent as shown in this study. Besides, our study also unearthed a slight increase in overall depression in re-positive patients, with some rated as normal or anxiety symptoms at initial-positive suffering from depression symptoms after re-positive. This result is consistent with previous studies in Wuhan (19). Patients receiving the RT-PCR re-positive result probably tend to suffer helplessness, worry, and disappointment, which facilitates the co-morbidity of anxiety symptoms and depression symptoms.

During the COVID-19 pandemic, health concerns are associated with clinically significant levels of psychological problems. WHO noted an increased risk of serious illness due to COVID-19 in patients with pre-existing non-communicable diseases, comprising cardiovascular disease, diabetes, cancer, etc. (20). Meanwhile, comorbid chronic disease has been identified as the most important risk factor for COVID-19 death (21), and some diseases such as myasthenia gravis may be exacerbated due to COVID-19 infection (22). Similar to previous studies (23–25), patients with other prior medical conditions were more likely to have anxiety symptoms. Besides, a significantly higher percentage of patients with a medical history in the re-positive group was also observed. These results may be attributed to the possibility that patients with medical history are more worried about their health status and fear and uncertainty about the physical impact of the interaction between the underlying disease and COVID-19 infection. Moreover, this distress and concern of patients with medical history may be aggravated after re-positive, triggering varying degrees of insomnia. Findings imply the significance of psychological interventions to address the heightened risk of depression and insomnia in re-positive COVID-19 patients.

This study had several impediments. First, the results of this study were limited to a sample of COVID-19 patients with non-severe disease types. Next, we employed a self-assessment scale, which may lead to recall bias. The current study points up the critical importance of screening and monitoring the psychological symptoms of re-positive COVID-19 patients and offering necessary psychological support and intervention. In this process, special attention should be paid to the anxiety symptoms of initial-positive COVID-19 patients, depression in re-positive COVID-19 patients, and insomnia symptoms of both initial and re-positive patients, to lessen the psychological burden of patients with COVID-19.

## Data availability statement

The original contributions presented in the study are included in the article/Supplementary material, further inquiries can be directed to the corresponding author/s.

## Ethics statement

This study was approved by the Research Ethics Committee of the First Affiliated Hospital of Nanchang University (Ethical number: 2020-051). The patients/participants provided their written informed consent to participate in this study.

## Author contributions

ML planned the study. XW and QF were involved in the data collection and curation process. HC performed the data processing. YH and TG analyzed the results and interpreted them. YL and JX drafted the original manuscript. HC and ML implemented major revisions to the manuscript and ultimately approved the manuscript for publication. All authors contributed to the article and approved the submitted version.

## Funding

This project was funded by the Science and Technology Plan Projects of Jiangxi Provincial Health Commission in 2020 (Grant: 2020-133); the Capital's Funds for Health Improvement and Research (Grant: 2022-2G-4116). The funding sources were not involved in the study's design, the collection, analysis, and interpretation of data, the writing of the report, and the decision to submit the article for publication.

## Conflict of interest

The authors declare that the research was conducted in the absence of any commercial or financial relationships that could be construed as a potential conflict of interest.

## Publisher's note

All claims expressed in this article are solely those of the authors and do not necessarily represent those of their affiliated organizations, or those of the publisher, the editors and the reviewers. Any product that may be evaluated in this article, or claim that may be made by its manufacturer, is not guaranteed or endorsed by the publisher.
