# Supplementary material for: The changes in psychological symptoms of COVID-19 patients after “re-positive”
Source: Front Psychiatry. 2022 Oct 10;13:1010004. doi: 10.3389/fpsyt.2022.1010004 (PMC9589489; doi:10.3389/fpsyt.2022.1010004)
Supplement: Supplementary file 1 [file Table_1.DOCX]

**Supplemental Table 1** The sociodemographic characteristics between 62 initial-positive cases and 32 re-positive cases

| Characteristics | Group | Initial-positive cases N (%) | Re-positive cases  N (%) | *P* |
| --- | --- | --- | --- | --- |
| Gender | Male | 29 (46.8%) | 10 (31.3%) | 0.187 |
|  | Female | 33 (53.2%) | 22 (68.7%) |  |
| Age | <45 | 35 (56.5%) | 12 (37.5%) | 0.063 |
|  | ≥45 | 27 (43.5%) | 20 (62.5%) |  |
| Medical history | No | 57 (91.9%) | 22 (68.7%) | **0.006** |
|  | Yes | 5 (8.1%) | 10 (31.3%) |  |
| The total duration of isolation, day | 7-15 | 13 (21.0%) | N/A | N/A |
|  | >15 | 49 (79.0%) | 32 (100.0%) |  |
| Marital status | Married | 48 (77.4%) | 24 (75.0%) | 0.802 |
|  | Others | 14 (22.6 %) | 8 (25.0%) |  |
| Educational level | High school or less | 51 (82.3%) | 27 (84.4%) | 1.000 |
|  | Undergraduate degree/college | 11 (17.7%) | 5 (15.6%) |  |
| Source of income | No | 16 (25.8%) | 7 (21.9%) | 0.315 |
|  | Not sure | 10 (16.1%) | 2 (6.2%) |  |
|  | Yes | 36 (58.1%) | 23 (71.9%) |  |
